# Microbial growth and physiology: a call for better craftsmanship

**DOI:** 10.3389/fmicb.2015.00287

**Published:** 2015-04-14

**Authors:** Thomas Egli

**Affiliations:** Environmental Microbiology, Swiss Federal Institute of Aquatic Science and Technology (Eawag),Dübendorf, Switzerland

**Keywords:** cultivation, batch, continuous culture, growth media, nutrient limitation, physiology

## Abstract

Virtually every microbiological experiment starts with the cultivation of microbes. Consequently, as originally pointed out by Monod (1949), handling microbial cultures is a fundamental methodology of microbiology and mastering different cultivation techniques should be part of every microbiologist’s craftsmanship. This is particularly important for research in microbial physiology, as the composition and behavior of microbes is strongly dependent on their growth environment. It has been pointed out repeatedly by eminent microbiologists that we should give more attention to the media and culturing conditions. However, this is obviously not adhered to with sufficient rigor as mistakes in basic cultivation principles are frequently found in the published research literature. The most frequent mistakes are the use of inappropriate growth media and little or no control of the specific growth rate, and some examples will be discussed here in detail. Therefore, this is a call for better microbiological craftsmanship when cultivating microbial cultures for physiological experiments. This call is not only addressed to researchers but it is probably even more important for the teaching of our discipline.

“The study of the growth of bacterial cultures does not constitute a specialized subject or branch of research: it is the basic method of Microbiology"

Monod (1949).

## Setting the Scene/Introduction

To most of us, the name S. J. Pirt stands for a quantitative approach to microbiology. In his legacy, “the yellow book” *“Principles of Microbe and Cell Cultivation”* (Pirt, 1975), Pirt provides an account consisting of basic concepts and mathematical descriptions of cultivation techniques and processes of cellular growth. Surprisingly for “experimentalists”, the book contains little explicit experimental data (in contrast to the book by Dean and Hinshelwood, 1966, that covers very similar aspects). It appears as if he wanted to emphasize the *“principles”* that would guide the reader and researcher toward sound investigations, rather than distracting them from the main messages with too many experimental data points. Pirt (1975) explicitly focussed on “extracellular” factors (comprising nutrient availability, temperature, pH etc.) that influence microbial growth behavior and cellular composition, and not on “intracellular” (genetic, biochemical, cytological) aspects. In this sense, Pirt’s (1975) *“Principles of Microbe and Cell Cultivation”* is a logical consequence of Monod’s (1949) crucial comment made in his influential review: *“The study of the growth of bacterial cultures does not constitute a specialized subject or branch of research: it is the basic method of Microbiology.”* In other words, understanding and controlling growth are compulsory parts of any microbiologist’s craftsmanship, as virtually all microbiological investigations begin with the cultivation of microbial cells.

During the first year of my Ph.D, whilst working on how to make methylotrophic yeasts fit for the production of single-cell protein, I luckily came across Dawson’s (1974) collection of original articles on microbial growth. Here, together with editorial comments pointing out the significance of individual articles, I found research papers, reviews and personal comments on basic, and often simple, questions concerning stoichiometry, kinetics, cultivation techniques, and physiology, written in unmatched clarity. Much later, when giving lecture and practical courses on microbial growth physiology and stress response to masters and Ph.D students, it became obvious to me that, for far too long, the teaching of the basic knowledge of essential techniques for cultivating microbial cells, of the advantages and disadvantages of different methods, and of the consequences on the experimental results obtained, had been neglected. This is mirrored by the fact that the commonly used microbiology text books cover this area in a superficial, inadequate way. In fact, we should teach our students to start with “*Materials and Methods,*” particularly the section on growth and cultivation, when reading a new paper. Only in this way one gets an impression of the quality of the data and how to rate and interpret a piece of work.

It should be pointed out that the issues raised here are not restricted to those few organisms that we microbiologists have always studied in the laboratory, such as for example *Escherichia coli* or baker’s yeast, but are valid for all (including multicellular) organisms, independent of their nutritional type of energy generation (phototrophic or chemotrophic) or carbon source usage (i.e., autotrophic or organotrophic). Moreover, the basic (mostly stoichiometric) points addressed below are valid also for microbial growth in nature and, therefore, they are relevant to biogeochemical issues, too.

It is with this background in mind that I write these comments (which for some may be considered to be rather a personal view) on the current state of experimentation in research on microbial physiology. When preparing this contribution I went back to some of the early literature and – somewhat to my surprise – found that many of the points addressed here had actually been critically referred to much earlier. Hence, it seems only fair to cite some of my “forerunners” literally. Unfortunately, it appears that during the last 50–60 years (since the time of Monod and Pirt) their messages have not reached the fertile ground they deserve. To me, this justifies another round of “whistle-blowing.”

## Looking Back

### “Classic” Microbial Growth Physiology

The rapid developments between 1930 and 1960 in genetic and biochemical techniques, in methods for controlled cultivation, and advances in the quantitative description of microbial growth, led to a true “harvest period” between 1940 and 1970 with respect to our understanding of microbial physiology (in terms of both accumulation of experimental data and development of concepts). This started after the introduction of the technique for cultivating submerged batch cultures in shake flasks, which allowed the basic principles of stoichiometry of nutrition and cellular composition to be confirmed and established experimentally. The invention of the continuous (chemostat) culture technique (the principle was already being used since the 1920s in chemical synthesis and engineering and fermentations, Haddon, 1928 see also Panikov, 1995) then allowed microbial populations at physiological states set by the investigator to be maintained over an extended period of time. This again permitted reproducible examination of fundamental physiological questions during growth under defined environmental conditions as a function of specific growth rate. The concept of balanced (“steady-state”) microbial growth was established (i.e., that in a microbial population the concentration of all cellular components increases at the same rate during growth under constant environmental conditions) and that some of these bulk components are dependent primarily on specific growth rate, not on the chemical composition, the complexity of the medium employed, or the cultivation system used (Herbert et al., 1956; Herbert, 1961). Furthermore, for the first time, good quality experimental data were produced that allowed a sound mathematical description of the kinetics of microbial growth as a function of the concentration of a single “limiting” (growth rate-restricting) nutrient (Powell, 1967).

Another milestone with respect to understanding how microbial cells respond to their environment through regulating their physiological activities was the discovery of coordinate gene expression of lactose-utilizing enzymes in *E. coli* in the mid-1950s by Jacques Monod and colleagues. This led to the identification of operons and regulons and their master regulatory compounds.

During this period microbial physiologists were driven by the desire to understand how a cell integrates the many reactions at the molecular and biochemical levels into a coordinated behavior that allows survival, successful competition for nutrients and hence proliferation. Experimental tools that allowed studies in “clean and well-defined systems” were definitely the starting point, but ultimately it was also about understanding a cell’s behavior in its natural habitat (see the recent comments by Neidhardt, 1999 and Schaechter, 2006). Outstanding accounts of the concepts and achievements of this time can be found in a fascinating collection of excellently written original articles, which also includes comments from the editor; this book should be basic reading for every microbiologist (Dawson, 1974). In addition, several text books were published at the end of this period (e.g., Dean and Hinshelwood, 1966; Kubitschek, 1970; Pirt, 1975; Ingraham et al., 1983).

### The Shift to Novel Molecular Methods: a Quantum Leap in (micro)biology

During the past 50 years we have witnessed an almost exponential development of molecular techniques and methods that allow the dissection and manipulation of cells and their components. This started in the mid-1950s with a method for sequencing proteins, followed some 20 years later by a technique that allowed the sequencing of DNA. Since the 1970s, various methods have been developed for manipulating and synthesizing DNA and RNA, and for separating, visualizing and quantifying other cellular constituents, notably proteins and metabolites. Many of these methods allow the totality of certain molecular species and events to be monitored. This offers the advantage of also combining “target”-directed analysis with a “non-target” screening of (bio)chemical species in microbial cells, cultures or even communities. In addition, the latter allows unanticipated aspects to be detected and for the system to be approached without pre-conceived ideas. The text book by Neidhardt et al. (1990) might be considered as an “early hybrid” of these two areas. In combination with the ability to handle and analyze large data sets with computational informatics, this allows access to genomes, transcriptomes, proteomes, or metabolomes, not only of microbial populations in laboratory cultures, but also of natural consortia, and probably in the near future even routinely at the single cell level.

## Revival of Microbial Physiology and the Art of Growing Cells; or “Déja-Vu”?

*Per se*, all of these advanced molecular tools, together with established biochemical methods, should allow today’s microbiologist to obtain an in-depth understanding, at all levels, of how microbial cells function and interact with their environment, from their biochemistry to cellular structures, entire cells, and even to the level of microbial consortia. Indeed, after an initial period that focussed mainly on method development (roughly from 1970 to 1990), employing “omics” in microbiological research has become routine. Often, similar questions are addressed today as were posed earlier in the heydays of classic microbial physiology (e.g., cellular composition during a batch growth cycle or responses to changing environmental conditions and stresses).

However, in many of the papers being published at present that are addressing physiological questions, I recognize a considerable imbalance in the attention given to molecular issues compared with that given to the media and cultivation conditions. It appears as if some fundamental principles and craftsmanship with respect to experimentation with microbial cultures (also individual cells) have been lost during the recent decades of method development “after Pirt.” Relating this to Monod’s (1949) statement, microbial growth seems to be considered as a “specialized subject,” a point that has little relevance to the question that one wants to investigate (or, alternatively, one assumes that “such simple issues” were sorted out long ago and that it is safe to follow “standard” or often used procedures). The phenomenon is not new, though. For example, in his preface, Dawson (1974) had already commented: *“In 1949 Monod observed that the study of bacterial growth was not in itself a specialized subject but a method basic to the discipline, an observation very true now for microbial growth as it relates to the wider field of microbiology. Perhaps it is because very few textbooks give growth more than a cursory mention that its basic significance is not generally recognized: for, regrettably, the fact that growth is indeed basic to the discipline goes largely ignored in the general practice of microbiology and related endeavors. The omission often leads to superficial experimentation and much wasted effort, thus cluttering the literature with a lot of meaningless data.”* This rather harsh statement, unfortunately, still holds today.

### Cellular Composition and Behavior Depends on and Varies with Growth Conditions

Central to this critical discussion is the fact that the composition of a microbial cell is strongly dependent on its growth conditions, in particular on the nature of the nutrient that stoichiometrically limits growth and on specific growth rate (μ). This holds true not only for all cellular constituents and metabolic pools but also for the elemental composition of the microbial biomass; the latter is particularly relevant when considering quantitative nutritional aspects. Herbert (1961) phrased this rather explicitly more than 50 years ago: *“There are few characteristics of micro-organisms which are so directly and so markedly affected by the environment as their chemical composition. So much is this the case that it is virtually meaningless to speak of the chemical composition of a micro-organism without at the same time specifying the environmental conditions that produced it. […] The extent to which chemical composition is affected by the environment has come to be realized only fairly recently, and a great deal of early work […] is unfortunately invalidated through lack of adequate environmental control.”*

Herbert’s (1961) statement is visualized in numbers in **Table 1**, where average values and reported ranges of variation are listed for both elemental composition and some major polymeric cellular constituents, together with examples of growth conditions that lead to such values. Whereas the cellular fraction of C, O, and H remains within a narrow range, the portion with the major nutrients N, P, S, K, and Mg may actually vary 3- to 10-fold. The fraction of trace elements can easily vary by two or more orders of magnitude (Egli, 2009). This group of nutrients includes iron, which is required in fairly large amounts by aerobic organisms (note that the yield factor used here for iron represents a “worst case” scenario). Its absence from catalytic centers of, e.g., cytochromes, catalases, or oxygenases, affects metabolic processes that involve oxygen in the broadest sense. Needless to say, a growth medium should contain all these components in sufficient amounts in order to ensure good healthy and reproducible growth of a microbial culture during all phases of cultivation.

**Table 1:** Average elemental composition (A) and content of major cellular polymeric constituents of microbial biomass (B), and range of variation with the corresponding environmental conditions (condensed from Egli, 2009).

(A)					
**Elemental constituents in dry biomass**	**Average^a^** (% **of DW)^b^**	**Range** (% **of DW)^b^**	**Average Y_**X/E**_ for C-limited growth**	**Excess factors recommended for C-limited medium**
C	50	45^c^–58^d^	1	Limiting
O	21	18^e^–31^f^	–	–
N	12	5^d^–17^g^	8	3–5
P	3	1.2^h^–10^i^	33	5–10
S	1	0.3–1.3	100	5–10
K	1	0.2^j^–5^k^	100	5–10
Mg	0.5	0.1^l^–1.1	200	5–10
Fe	0.5	0.01–0.5	200	10–20

(B)

**Cellular constituents**	**Average^a^** (% **of DW)^b^**	**Range** (% **of DW)^b^**	**Cellular constituents**	**Average^a^** (% **of DW)^b^**	**Range** (% **of DW)^b^**

Protein	55	15^m^–75	Phospholipids	9	0^s^–15
RNA^n^	21	5^m^–30^o^	Glycogen	3	0–50^t^
DNA^n^	3	1^m^–5^p^	PHB	–	0–80^t^
Peptidoglycan	3	0^q^–20^r^	Polyphosphate^n^	–	0–20^u^

*DW, dry weight, PHB, polyhydroxybutyrate; growth yield factor, Y_X/E_ (_g_ DW/g element); ^a^Gram-negative cells growing with excess of all nutrients at μ_max_ in batch culture; ^b^cells contain on average 70% of water; ^c^C-limited cells containing no reserve materials; ^d^N-limited cells storing PHA or glycogen; ^e^cells grown N-limited with neutral lipids; fcells grown N-limited accumulating glycogen; ^g^cells growing at high μ containing high levels of RNA; ^h^cells grown P-limited; ^i^cells accumulating polyphosphate; ^j^Gram-positive Bacillus spores; ^k^Gram-positive bacilli; lMg-limited cells at low μ; ^m^cells storing carbonaceous reserve materials; ^n^inclusion of highly negatively charged polymers is paralleled by appropriate amounts of counter-cations, usually Mg^++^, Ca^++^, or K^+^; ^o^at high specific growth rates; ^p^cells growing slowly; ^q^parasitic cell wall-less species; ^r^Gram-positive bacteria; ^s^strains replacing phospholipids with P-free analogs under P-limited conditions; ^t^cells grown N-limited; usome yeast and bacteria*

## Craftsmanship in Cultivation: Some Facts on What Too Often Goes Wrong

With respect to craftsmanship and the art of culturing microbial cells there are two points, which – in the author’s eyes – seem prone to basic mistakes and, therefore, should receive more attention. The first point concerns the use of (in)appropriate media, and includes simple stoichiometric issues of nutrition; this point is independent of the method employed for culturing cells, i.e., it applies for both batch and continuous cultivation systems. The second point is a certain lack of control of specific growth rate over a sufficiently long time to perform experiments under balanced growth conditions; this point applies in particular to experiments performed in batch culture. This contribution will focus on these two points only, although a number of other points might also need more attention.

### The Growth Medium: is it Appropriate and Does it Serve the Purpose?

#### Medium Composition: Quantitative Aspects

With respect to quantitative aspects, growth yield factors for elements, Y_X/E_, can be derived from the elemental composition of the biomass. They provide a stoichiometric link between the amount of a nutrient supplied in a medium and the biomass formed from it. The first growth yield factors were actually reported by Raulin, a student and collaborator of Pasteur (cited in Pirt, 1975), and these factors have a long tradition and have been confirmed for many different organisms. They can be applied for the design, analysis and optimization of media used for cultivation of bacterial cultures, including molds, yeasts, algae, and protozoa (Pirt, 1975; Egli, 2009). Approximate yield factors for the quantitatively most relevant elements are listed in **Table 1**. Growth yield factors can be defined not only for nutrients that are built into the biomass, but also for electron donors and acceptors that serve as energy sources and terminal electron acceptors (Egli, 2009). Yields vary according to cultivation conditions and those listed in **Table 1** are average values relevant for carbon-limited growth. For example, when growing C-limited, microbes usually produce 8 g of dry biomass from 1 g of nitrogen; however, if the nitrogen source is limiting growth and carbon plus all other nutrients are in excess, many organisms are able to store excess available carbon in the form of intracellular reduced carbonaceous reserve materials, such as glycogen or PHB. Under such conditions, the Y_X/N_ can reach 20 or more grams of dry biomass per gram of nitrogen.

#### A Simple and Quick “Soundness-Check” for Growth Media

When assessing appropriateness of a medium for an experiment, the first question concerns that of the nutrient stoichiometrically limiting growth in the cultivation system. This nutrient will determine the maximum concentration of the biomass in the system (most commonly, for heterotrophs it is the carbon/energy source). This nutrient determines the requirement for all other sources of nutrients that have to be supplied in the medium. In a good medium an excess of all non-limiting nutrients is supplied and typical excess factors are also listed in **Table 1**.

The growth yield factors given in **Table 1** can be used to design and to analyze mineral media. For simplicity, we concentrate here on defined mineral media to illustrate some of the quantitatively most critical points (the case of complex media will be treated later). An example is given in **Table 2** for a medium that has been widely used for cultivating *E. coli* in the research literature published over the last 50 years. An “improved version” of medium M9 is used here (e.g., Sambrook et al., 1989), as is also listed in many microbiological text books (e.g., Madigan et al., 2012); additionally, it is similar to the commercially available “M9 Minimal Salts”-based medium. The calculated excess factors in **Table 2**, indicate that when supplemented with 4 g/L of glucose, the nitrogen available in the medium is slightly in excess. However, if 10 g/L of glucose are added (as is done frequently and suggested in Madigan et al., 2012) the medium is carbon-deficient and nitrogen – judging by the bulk nutrients alone – is now the stoichiometrically limiting nutrient. Phosphorus, potassium and sulfur are supplied in large excess due to the buffering system used, whereas magnesium is most probably not present in sufficient excess. Availability of all trace elements, in particular of iron, is theoretically severely restricted in this medium. Hence, when considering the M9-based medium given in **Table 2** addition of nitrogen, magnesium and of trace elements is necessary to obtain a medium that is clearly limited by the availability of the carbon/energy source (or, alternatively, a corresponding reduction in the concentration of glucose that brings all other nutrients into excess). This simple “on the back of an envelope” calculation suggests that the frequently used M9-medium is not ideal for defined physiological studies.

**Table 2:** Composition and analysis of defined minimal medium M9, which is regularly employed for carbon/energy-limited cultivation of *Escherichia coli* strains with glucose or other carbon/energy sources.

Nutrient	Medium component	Amount (g/L)	Amount element (g/L)	Yield factor (g DW/g element)	DW predicted (g/L)	Excess factor over C
C	Glucose, C_6_H_12_O_6_	4 up to 10	1.6 up to 4.0	1	1.6 up to 4.0	1 (limiting by definition)
N	(NH_4_)_2_SO_4_	1.0	0.21	8	1.7	1.06^∗^–0.42^∗∗^
P	K_2_HPO_4_, KH_2_PO_4_	7.0 2.0	1.71	33	56.4	35^∗^–14.1^∗∗^
S	(NH_4_)_2_SO_4,_ MgSO_4_	1.0 0.1	0.242	100	24.2	15^∗^–6^∗∗^
K	K_2_HPO_4_, KH_2_PO_4_	7.0 2.0	3.71	100	371	232^∗^–93^∗∗^
Mg	MgSO_4_	0.1	0.202	200	4.0	2.5^∗^–1.0^∗∗^
Ca	CaCl_2_	0.002	0.00072	100	0.072	0.05^∗^–0.02^∗∗^
Trace elements	Fe, Co, Mn, Zn, Cu, Ni, Mo	each at 2–10^•^10^-6^	Fe: 0.00001 Mn: 0.00001	Fe: 200 Mn: 10’000	Fe: 0.002 Mn: 0.1	for Fe: 0.0013^∗^–0.0005^∗∗^ Mn: 0.06^∗^–0.025^∗∗^

*Slight variations of this medium, either in the type, ratio and concentration of the buffering phosphate salts, the use of ammonium chloride instead of ammonium sulfate, or without addition of Ca or trace elements, can be found in the literature; however, all these variations do not change the presented analysis significantly. The medium composition listed here was taken from (Madigan et al., 2012). For trace elements, only the availability of iron and manganese was considered in this table assuming addition of 10 μg/L, each. ^∗^ assuming 4 g/L of glucose; ^∗∗^ assuming 10 g/L of glucose. Gray boxes indicate medium constituents that are predicted to be of limited availability*.

In favor of “established media that have always been used,” it should be indicated that not so long ago, many of the bulk components used for preparing media, e.g., those used for pH buffering, were only of technical grade, and often tap water was used. Thus, trace elements in particular were supplied as impurities rather than in defined amounts. Hence, some of the “traditional” media might have performed better than they do today where the quality of components used for medium preparation are of much higher purity. However, such simple media might actually be sufficient in non-physiological experiments for “producing biomass” for isolation of cellular components that are not affected by nutrient-deficiencies or limitations such as, for example, isolation of DNA for sequencing (see Sambrook et al., 1989).

#### Experimental Verification

**FIGURE 1:**
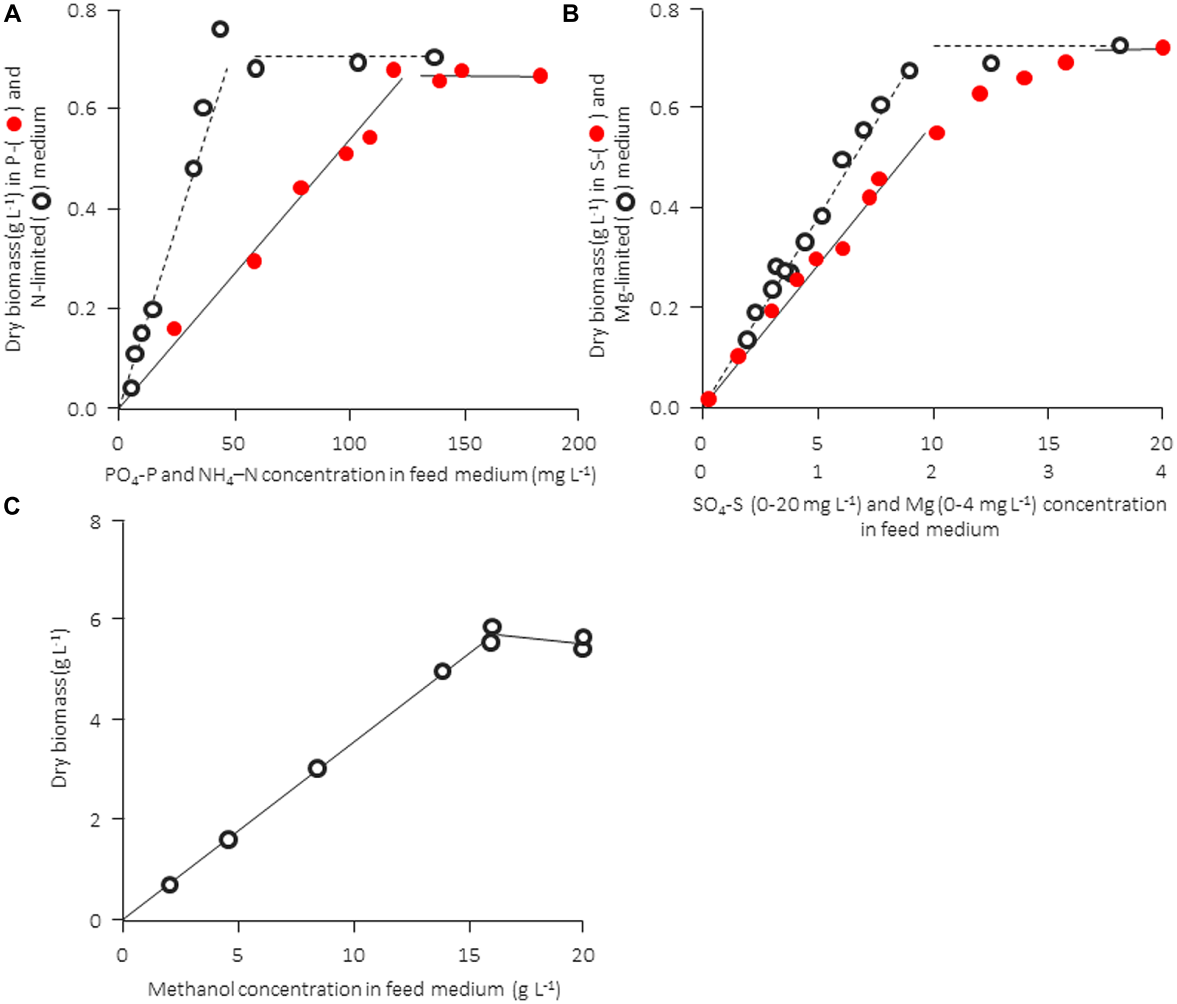
Experimental confirmation of stoichiometric limitation of growth of different nutrients for a bacterial (Cometta et al., 1982) and a yeast culture (from Egli, 1980). Both microbial strains were cultivated in a chemostat culture at a fixed dilution rate. A distinct transition from limitation to excess is observed for carbon-, nitrogen, and phosphorus-limited growth, whereas the transition between limitation and excess is not as distinct in the case of magnesium and sulfur. **(A,B)** Rearranged from Cometta et al. (1982), and information kindly provided by B. Sonnleitner. **(C)** Rearranged from Egli (1980).

The computational analysis in Table 2 is not “purely theoretical” but it is based on much experimental evidence. In practice, however, there are a number of additional factors that influence the quality and performance of a medium (pH, procedure of sterilization or temperature of storage, and occasionally also other unknown factors). Therefore, it is useful to confirm the growth-limiting factor for a given experimental setup with hard data. The nature of a growth-limiting nutrient in a particular medium and the growth yield factor can be confirmed experimentally in a straightforward way, namely by determining the concentration of biomass formed as a function of the concentration of a particular nutrient (keeping all other medium components unchanged). This can be done either in batch experiments (measuring the final concentration of dry biomass formed) or in continuous culture at a constant dilution rate (monitoring the steady-state dry biomass concentration). Examples are shown in **Figure 1** for a bacterial and a yeast strain. The concentration of the produced biomass must be strictly proportional to the concentration of the limiting nutrient and the straight line should pass through the origin; if this is not the case, biomass formation is influenced by one or more (unknown) additional factors [discussed in Egli (1991) and Egli (2009)]. Also, in most of the examples given in **Figure 1** the data suggest a clear-cut transition from nutrient-limited to non-limited conditions. The graph for the sulfur source, where the transition between the horizontal and the proportional growth region appears to occur gradually rather than abruptly, may indicate a more complex interaction of two or maybe even more factors simultaneously (see e.g., Egli, 1991) and suggests that care has to be taken not to run into this region (see Some Remarks on Chemostat Cultivation). Unfortunately, such basic tests are rarely carried out or reported, and many examples of the use of media that do not satisfy the basic requirements discussed above can be found in the literature. As a rule of thumb, in the author’s laboratory we would cultivate at approximately one third of a medium’s theoretical carrying capacity at an intermediate specific growth rate (i.e., roughly μ_max_/2). This allows flexibility and a sufficient excess of all nutrients, also during growth close to or at μ_max_ in batch culture, without having to change the medium composition.

**FIGURE 2:**
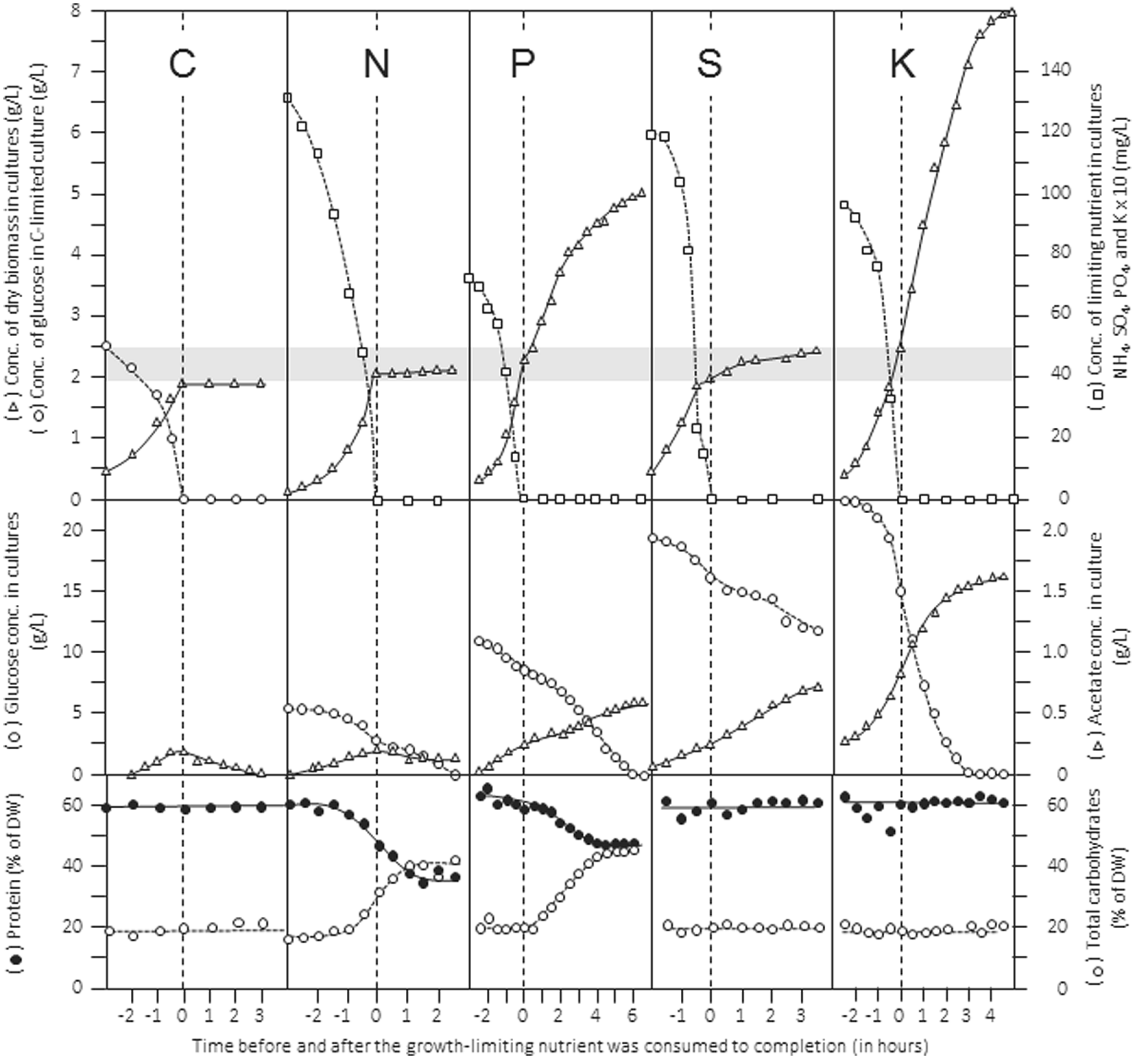
Patterns of growth, nutrient consumption, product formation and gross cellular composition during batch growth of *Klebsiella pneumoniae* in a defined mineral medium limited by different nutrients. The limiting nutrients were carbon (glucose), nitrogen (NH_4_^+^), phosphorus (PO_4_^3-^), sulfur (SO_4_^2-^), and potassium (K^+^), respectively. Top panels show concentrations of dry biomass and limiting nutrient; middle panels show concentrations of residual glucose and acetate formed in the medium; the bottom panels show composition of biomass with respect to total protein and carbohydrates. Rearranged from Wanner and Egli (1990).

An extended review of how stoichiometric limitation of different types of nutrients affects gross composition and physiology of microbial cells during batch cultivation was published some time ago (Wanner and Egli, 1990). The patterns of batch growth curves, dynamics of cellular composition, substrate utilization and product formation are shown in **Figure 2** for a culture of *Klebsiella pneumoniae* cultivated in the same defined mineral medium but limited by either the source of carbon (glucose), nitrogen (ammonia), phosphorus (phosphate), sulfur (sulfate), or potassium (K). In this example, the initial concentration of the limiting nutrient in the medium was chosen such that it should support the formation of 2 g L^-1^ of dry biomass (indicated by the gray band). The data give an impression of the importance for ensuring cultivation under explicitly known nutritional conditions and support the elemental yield factors listed in Table 1. They also point to the effects of restricted nutrient availability, and visualize the effect of shifts in the nature of the limiting nutrient during batch cultivation on the growth behavior of microbial cultures. For example, judged from the pattern of the biomass concentration, the transition from C-excess to C-limitation occurs abruptly, whereas the transition from K-excess to K-limitation is not obvious and can be assessed only from the experimentally determined K-concentrations in the medium. In all cases, consumption of excess glucose continued at a similar rate after the different nutrients became limiting. At the same time, acetate production from glucose continued; only under C/energy-limited conditions acetate produced in the unlimited growth phase was consumed after exhaustion of glucose. Furthermore, the data visualize the importance of adding sufficient C/energy source because in three cases glucose, despite being initially supplied in considerable excess, was consumed to completion, resulting in the end of cells physiologically limited simultaneously by two nutrients, which is particularly obvious in the K-limited batch culture. In the S-limited data set, the pattern recorded for the concentrations of remaining sulfate and produced dry biomass suggests the transition from a low-affinity to a high-affinity transport system.

### Control of Specific Growth Rate at Balanced Growth

The second important parameter that influences the physiological state and cellular composition is the specific growth rate (μ, h^-1^). The strong dependence on μ of cellular composition with respect to “bulk components” (i.e., protein, RNA, DNA) was established in the heydays of microbial growth physiology (Herbert, 1961). It is impressive to see that data for the cellular composition as a function of μ obtained from a glucose-limited chemostat perfectly match those from cells growing at μ_max_ in batch cultures performed with different carbon sources in order to vary μ (Ingraham et al., 1983), demonstrating dependence of these constituents on μ only and not on the method used for cultivation. Ever since then, the link between μ and gross cellular composition, regulation of gene expression, of cellular concentrations of regulators and resulting metabolite pools have remained a fascinating, open area for research (e.g., Schaechter, 2006; Klumpp et al., 2009; Klumpp and Hwa, 2014).

Both batch and continuous culture systems are commonly employed in order to obtain cells for investigating the influence of μ on microbial behavior. Although the two concepts of cultivation are different, both allow environmental conditions for the culture to be maintained to ensure balanced growth conditions long enough to perform physiological experiments – if used properly.

#### Some Remarks on Chemostat Cultivation

The technique of continuous cultivation allows (single-celled) organisms to be cultured under balanced growth conditions over virtually the whole range of specific growth rates without changing the medium composition, by simply setting the dilution rate, D. The quality of the equipment available today makes it possible to set D with such precision that it is possible to achieve steady-state growth conditions at virtually any desired μ between 0< μ <μ_max_. If the strain used “behaves well” (e.g., does not stick to surfaces or does not form aggregates), stability can be maintained for weeks and the experiments become “time-independent,” so that analyses can be repeated again and again. In this system, specific growth rate μ is controlled by the *in situ* concentration of the growth-limiting nutrient, which, at the same time, exerts a stoichiometric limitation and determines the concentration of biomass in the system (Herbert et al., 1956; Pirt, 1975).

An important advantage of chemostat cultivation is that cell density has – in theory – no effect on the physiological state as long as the limitation regime remains the same (Herbert et al., 1956). This was experimentally demonstrated for *E. coli* for both kinetic and physiological properties (Senn et al., 1994; Ihssen and Egli, 2004). However, recently it was found that there are exceptions to the rule, because cell density was shown to influence the speed of selection for mutants with improved transport affinity for the growth-limiting nutrient (Wick et al., 2002). Nevertheless, in general, biomass concentrations in a chemostat culture can be adjusted according to analytical needs with no effect on the cellular physiology. Usually, very low cell densities are favorable for investigating growth kinetics (Senn et al., 1994; Kovarova-Kovar and Egli, 1998), whereas high biomass concentrations are used for acquiring good data for properties for which only insensitive analytical methods are available.

**FIGURE 3:**
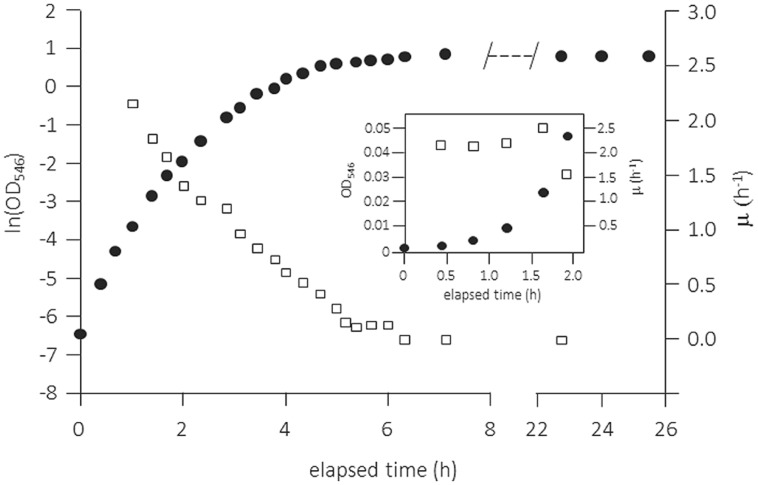
Zones of single- and dual-nutrient-limited growth (gray area) for a culture of *Klebsiella pneumoniae* cultivated in the chemostat as a function of dilution rate and C:N-ratio in the feed medium. **(A)** Conceptual scheme of residual steady-state concentrations for the nitrogen (ammonia) and the carbon source (glycerol) at a dilution rate (D) of 0.1 h^-1^, as well as the steady-state biomass concentration in the culture and of an accumulated reserve material. In this experiment, the concentration of the nitrogen source in the feed medium is kept constant and the C:N-ratio of the medium is varied by changing the concentration of the carbon source). **(B)** The predicted boundaries for the three growth zones are shown, the boundaries were calculated from literature data reported (see Egli, 1991). The left hand border (full circles) was calculated from reported growth yields for N and C determined under glycerol-limited growth conditions; the right hand border (empty circles) was calculated from growth yields reported from cultures grown under ammonia-limited growth conditions. Adapted and extended from Egli (1991).

Inappropriate use of continuous culture, however, may result from the use of unbalanced media that are not distinctly limited, or in which the growth-limiting factor shifts along with changes in the dilution rate. How this may happen and what the consequences are is visualized in the conceptual scheme in **Figure 3A**, which is linked with actual experimental data extracted from several reports on growth of *Klebsiella pneumonia* in chemostat culture with glycerol and ammonia as C- and N-source, respectively (**Figure 3B**). In this example, a strain is cultivated in a chemostat at a fixed D and biomass production is studied as a function of the C:N ratio of the inflowing medium. In **Figure 3A** we assume that the concentration of the N-source is kept constant whereas that of the C-source is increased stepwise from left to right. The resulting patterns for the steady-state concentrations of dry biomass (*x)*, residual N- and C-source (*n*, *c*), as well as the cellular content of reserve material is shown. During growth with media with a low C:N-ratio, i.e., with media that are C-limited, *x* produced increases linearly when more and more of the growth-limiting carbon source is added (compare **Figure 1**). Accordingly, more of the nitrogen source supplied will be used to produce biomass and, consequently, *n* decreases with increasing C:N-ratios of the inflowing medium. When a medium is fed with a C:N-ratio of ∼8, sufficient carbon is added to consume all of the nitrogen. Upon further addition of carbon, the culture is expected to grow N-limited and excess carbon to accumulate in the culture broth. However, by most organisms the surplus carbon is not left unused in the medium but is stored intracellulary in the form of reserves (PHB/A, glycogen, lipids, depending on the organism and the carbon source), for this reason, biomass continues to increase with increasing medium C:N-ratios. Left-over carbon starts to accumulate only when the reserve pools are filled up to the rim (here at a medium C:N > 16). The consequence, of this behavior is the existence of a large intermediate zone in which cells exhibit stable, balanced growth but are simultaneously limited by C and N. Such patterns were observed for a range of different bacteria and yeast cultures (see Egli, 1991, 2009).

The boundaries of zones can be predicted easily from growth yield factors determined for the two nutrients under either C-, or clearly N-limited conditions: The concentration of dry biomass formed in the culture under steady-state conditions (*x*) can be calculated based on either carbon or based on nitrogen (equation 1) from the feed concentrations of C and N (c_0_, n_0_), the actual steady-state concentrations (*c*, *n*), and the growth yields based on either C or N (Y_X/C_, Y_X/N_). Because both *c* and *n* are close to zero under C/N-limited growth conditions, equation 1 can be rearranged to give the C/N-ratio in the inflowing medium (c_0_/n_0_).

$$x=\left(c_{0}-c\right)Y_{X/C}=(n_{0}-n)Y_{X/N}\,\;\;\;\;\;\;\left(1\right)$$

$$c_{0}/n_{0}\,\;\approx Y_{X/C}=Y_{X/C}\,\;\;\;\;\;\;\;\;\;\;\;\;\left(2\right)$$

Growth yields for C and N are not constant but depend of nutrient limitation and μ; therefore, knowing μ and limitation-dependent Y_X/C_ and Y_X/N_ for a given strain/nutrient-set allows to determine the position of the boundaries of the three growth regimes. The left-hand boundary can be predicted from yields determined under C-limited conditions, whereas the position of the right hand border needs yields from N-limited cultures (for more see Egli, 1991).

Such a data set was extracted from the literature for *K. pneumophila* cultivated in the chemostat at different dilution rates with glycerol and ammonia (Egli, 1991) and the consequences are visualized in **Figure 3B** (other data sets exist also for a range of different bacteria and yeasts, see Egli, 1991, 2009). Extension and location of this C/N-limited zone is dependent on μ (**Figure 3B**) and available data demonstrate that it is particularly wide during slow growth (Egli, 1991). The “banana” shape of the C/N-limited zone is particularly sensitive on μ; its shift to higher medium C/N ratios is mainly influenced by the cells needs for maintenance energy requirements, resulting in reduced yields for carbon at low specific growth rates.

It should be pointed out that any change in growth yields, may it result from either intracellular accumulation of reserve material, or an excretion of overflow metabolites such as ethanol or acetate, will result in such “in-between-zones” between clearly, single-nutrient limited zones of growth. Growth in “in-between-zones” influences the cellular physiology and cells with “hybrid” composition can be obtained. Hence, broad “in-between-zones” can be expected for those nutrients of which the cellular content is reported to vary over a large range, such as P, S, or Fe (compare Table 1A). Unfortunately, so far only few systems concerning this phenomenon have been investigated in detail.

That changes in the nutritional growth regime can occur “unexpectedly” is indicated in **Figure 3B** with the double-headed arrow. In this example, the *K. pneumoniae* culture can be shifted from a C-limited to a N-limited growth regime and *vice versa* by a simple change in μ, even though the composition of the medium remained unaltered at a C:N-ratio of ∼7. Sifts of this type may occur quite frequently during shifts in specific growth rate; they can occur not only in continuous but also during transient situations in continuous and batch cultivation systems, as well as in ecosystems (Egli, 1995; Egli and Zinn, 2003; Zinn et al., 2011).

#### Some Remarks on Batch Cultivation

##### Defined mineral media

*Per se*, batch cultivation is based on growth at excess concentrations of all nutrients with (ideally) one of them limiting growth stoichiometrically. In mineral media used for cultivating heterotrophs, the carbon/energy source is usually selected as the growth-limiting nutrient, with concentrations of the growth-limiting nutrient being typically in the range of grams per liter (see A Simple and Quick “Soundness-Check” for Growth Media). In such media cultures, growth at μ_max_ is possible until the limiting nutrient has been consumed down to levels where it also affects μ kinetically (assuming Monod kinetics apply, this is roughly at concentrations corresponding to 10-times K_s_, hence, for *E. coli* growing on glucose, at less than a few mg/L). Cells for controlled physiological experiments are harvested from the exponential growth phase before kinetic restriction begins. With the concentrations used, the change from exponential growth at μ_max_ to stationary phase should be abrupt (see **Figure 2** for glucose-limited growth of *K. pneumoniae*) and not gradual. To obtain cells in balanced conditions they should have been growing for more than four doubling times at μ_max_; low-size inoculi from cultures already growing in the very medium at μ_max_ help to avoid lag-phases and “unbalanced” cell material. Unfortunately, lag periods before growth commences can be deduced from many of the published growth curves, in addition, there is frequently no information provided on this part of the experiment.

Batch cultivation, as conventionally performed, does not allow cells to be grown at different growth rates without changing the composition or complexity of the medium. The most common way of obtaining cultures growing exponentially with different μ_max_ is to change the nature of the limiting nutrient (for example, using acetate instead of glucose). This, however, will affect the cell’s growth physiology and it remains to be determined whether this is due to the change in specific growth rate or to the change of a medium component. With this approach, using 22 different media, Schaechter et al. (1958) investigated the dependence of cell size on specific growth rate in *Salmonella*.

Nevertheless, an unconventional, although tedious, procedure allows cells to be cultivated in the same medium under batch conditions at different specific growth rates. The trick is to lower the concentrations of the limiting substrate down into the K_s_ range. In order to circumvent significant changes in the concentration of the growth-rate-limiting substrate due to consumption, frequent sub-culturing of the cells in this medium is necessary. Such experiments require very clean water containing virtually no traces of contaminants that would interfere with the limiting nutrient and, hence, only allows experiments at very low biomass concentrations. The only example I am aware of are the experiments performed with *E. coli* cultures by Shehata and Marr (1971), who determined the Monod K_s_-constant for glucose in this way. In essence this technique can be considered a “discontinuous chemostat culture” with a repeated dilution at infinitesimal steps. Although the technique allows to work only at very low biomass concentrations (in the range of 0.1–1 mg dry weight/L, depending on the K_s_ for the limiting nutrient), the generally increased sensitivity of bioanalytical techniques may soon give access to a range of physiological parameters in such low density cultures.

##### Complex media

**FIGURE 4:**
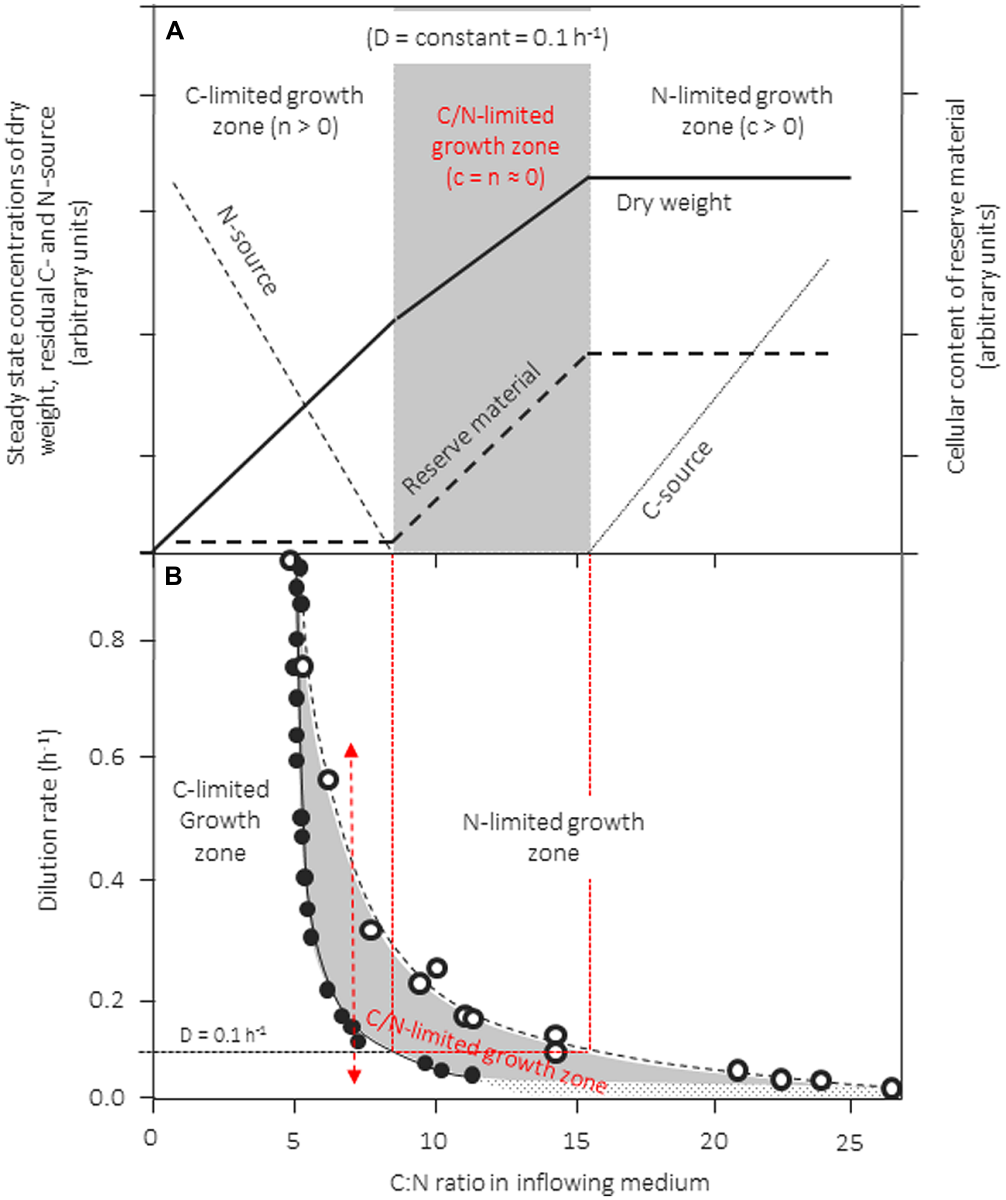
Batch growth curve of *Escherichia coli* K-12 MG1655 in complex medium (Luria-Bertani broth, 25% original strength) in vigorously shaken flask. Growth was measured as OD_546_, the temperature was maintained at 37°C, and pO_2_ was always >70% air saturation. The specific growth rate μ (h^-1^) was calculated as the slope of five adjacent points (empty squares). The inset shows the first 2 h of the experiment with μ determined from the slope of three adjacent points. Adapted from Berney et al. (2006) and amended with the inset. For more details see Berney et al. (2006).

The use of complex media, in particular of Luria-Bertani broth [(originally “lysogeny broth” (LB)] has always been very popular for cultivating (in particular heterotrophic) microbes. Although widely used and very convenient for the researcher, this method has many severe disadvantages when employed for physiological studies in batch culture. LB medium contains a digest of casein plus yeast extract along with small amounts of single amino acids, mainly peptides of varying lengths, and only small amounts of carbohydrates. In contrast to the common assumption, the medium supports only limited exponential growth and this only in the very early phase after low inoculation. An example is shown in **Figure 4** for the growth of *E. coli* in a shake flask but under fully aerobic conditions (Berney et al., 2006). Closer examination of the data in the very first phase right after inoculation suggests the presence of a short exponential growth phase during the first 2 h. This phase was only clearly visible when the inoculum size was low and the medium was filter-sterilized (heat-sterilized LB broth supported only lower specific growth rates throughout the growth cycle). After this short phase of exponential growth, μ decreased progressively. Cleary, throughout a batch growth cycle in LB medium the quality of the growth substrates changes permanently and cells have to adjust their physiological properties not only to the changing growth rate but also to ongoing alterations in the type and quality of the nutrients that are available for growth (see for example Baev et al., 2006).

Such data for the growth of *E. coli* and many other enterobacteria with LB medium have been produced over many years and large numbers of physiological studies are available in the literature. However, it is clear that LB has to be used with much more awareness and care as it neither allows control of the specific growth rate nor enables physiological studies to be performed under balanced growth conditions. Therefore, LB batch cultivation is a not an option when one wants to produce results that are reproducible and comparable to data obtained by other researchers. The recently published data for batch cultivation of an *E. coli* strain with TSB medium demonstrate virtually the same growth pattern as with LB (Lindqvist and Barmark, 2014): therefore, the remarks made above can be applied generally for complex media: They do not allow constant growth conditions (i.e., balanced growth in all respects) over a reasonable range of time. So, cultivation of cells in batch culture with complex media is in reality a complex way of culturing cells and variable or even irreproducible physiological data are a logical result.

## Final Comment

Investigations of microbial behavior usually begins with observations, brilliant ideas or concepts, which then have to be distilled into practical experiments that can (a) be performed, and (b) will yield results based on which we can proceed. This process has to be underpinned with solid microbiological work in the laboratory and on the microbial physiology. This should also include aspects of media and cell cultivation, preferably at an early stage of an experiment. If the craftsmanship we use is not sufficiently solid in any of the experimental steps we will produce low quality data, even though the intellectual idea might be excellent. Misleading concepts will disappear sooner or later, but incorrect data, once they have been published, are difficult to eliminate from the scientific literature because experiments are rarely repeated (I don’t know who said this, however, there is a lot of truth in it).

## Conflict of Interest Statement

The author declares that the research was conducted in the absence of any commercial or financial relationships that could be construed as a potential conflict of interest.
